# Genetic deletion of osteopontin in TRAMP mice skews prostate carcinogenesis from adenocarcinoma to aggressive human-like neuroendocrine cancers

**DOI:** 10.18632/oncotarget.6678

**Published:** 2015-12-19

**Authors:** Giorgio Mauri, Elena Jachetti, Barbara Comuzzi, Matteo Dugo, Ivano Arioli, Silvia Miotti, Sabina Sangaletti, Emma Di Carlo, Claudio Tripodo, Mario P. Colombo

**Affiliations:** ^1^ Molecular Immunology Unit, Department of Experimental Oncology and Molecular Medicine, Fondazione IRCCS Istituto Nazionale Tumori, 20133, Milano, Italy; ^2^ Functional Genomics and Bioinformatics, Department of Experimental Oncology and Molecular Medicine, Fondazione IRCCS Istituto Nazionale Tumori, 20133, Milano, Italy; ^3^ Department of Medicine and Science of Aging, Section of Anatomic Pathology and Molecular Medicine, “G. d'Annunzio” University, 66100, Chieti, Italy; ^4^ Ce.S.I. Aging Research Center, “G. d'Annunzio” University Foundation, 66100, Chieti, Italy; ^5^ Tumor Immunology Unit, Department of Health Sciences, University of Palermo, 90127, Palermo, Italy

**Keywords:** prostate cancer, extracellular matrix, osteopontin, neuroendocrine

## Abstract

Osteopontin (OPN) is a secreted glycoprotein, that belongs to the non-structural extracellular matrix (ECM), and its over expression in human prostate cancer has been associated with disease progression, androgen independence and metastatic ability. Nevertheless, the pathophysiology of OPN in prostate tumorigenesis has never been studied. We crossed TRansgenic Adenocarcinoma of the Mouse Prostate (TRAMP) mice with OPN deficient (OPN^−/−^) mice and followed tumor onset and progression in these double mutants. Ultrasound examination detected the early onset of a rapidly growing, homogeneous and spherical tumor in about 60% of OPN^−/−^ TRAMP mice. Such neoplasms seldom occurred in parental TRAMP mice otherwise prone to adenocarcinomas and were characterized for being androgen receptor negative, highly proliferative and endowed with neuroendocrine (NE) features. Gene expression profiling showed up-regulation of genes involved in tumor progression, cell cycle and neuronal differentiation in OPN-deficient versus wild type TRAMP tumors. Down-regulated genes included key genes of TGFa pathway, including SMAD3 and Filamin, which were confirmed at the protein level. Furthermore, NE genes and particularly those characterizing early prostatic lesions of OPN-deficient mice were found to correlate with those of human prostate NE tumours. These data underscore a novel role of OPN in the early stages of prostate cancer growth, protecting against the development of aggressive NE tumors.

## INTRODUCTION

Prostate carcinoma (PCa) is one of the most common tumors in developed countries and the second leading cause of cancer-related death in the USA [1]. PCa is a multifocal disease, which progresses from high-grade prostate intraepithelial neoplasia (PIN). Initially, the tumor responds to therapies that mainly consist in androgen ablation, but it eventually becomes androgen resistant due to alterations in androgen receptor expression or related signalling. The main drivers of PCa development and progression are still unknown and effective therapies for the androgen-resistant stage are not yet available.

Stromal microenvironment modifications occurring at the stage of precancerous PIN lesions are no longer considered bystanders. In this context, SIBLINGs (Small Integrin-Binding Ligand N-Linked Glycoproteins), a family of non-structural extracellular matrix (ECM) proteins, seem to favor tumor progression by hijacking their physiological functions in wound healing and tissue remodeling [2].

Osteopontin (OPN) is a secreted protein, belonging to SIBLINGs, present in body fluids and almost ubiquitous in tissues. OPN contributes to tissue homeostasis through the regulation of stem cell pools within specific niches [3]. OPN over-expression in human PCa has been associated with disease progression towards androgen independence and metastasis [4, 5]. Therefore, at the late stages of PCa OPN targeted inactivation might be a promising strategy. However, the role of OPN at onset of PCa has not been investigated so far. To address this issue, we crossed OPN deficient (OPN^−/−^) mice with the TRansgenic Adenocarcinoma of the Mouse Prostate (TRAMP) mice, which carry the SV40 large T antigen (Tag) under the control of the rat probasin regulatory element, selectively activated by androgens in the prostate epithelia. TRAMP mice invariantly develop PIN (8-16 weeks) that progresses to focal invasive adenocarcinoma (16-20 weeks) and then to moderately differentiated (24 weeks) and poorly differentiated (24-30 weeks) adenocarcinoma [6, 7].

Although the reported over-expression of OPN in advanced disease let envisage tumor reduction in OPN^−/−^TRAMP mice, we unexpectedly observed the early onset of highly proliferative, anaplastic androgen-independent tumors, characterized by a neuroendocrine (NE) phenotype. The corresponding human NE carcinoma characterized by aggressive behaviour, poor response to standard treatments and dismal prognosis [8, 9] accounts for nearly 10% of cases and seldom occurs in relatively young patients. Notably, areas of NE differentiation occur in advanced PCa patients after androgen ablation therapy [10, 11]. Five to ten percent of TRAMP mice spontaneously develop tumors with the NE phenotype. The mechanisms driving their onset in OPN deficiency are here investigated [12].

## RESULTS

### OPN deletion enhances anaplastic tumor development in TRAMP mice

**Figure 1:**
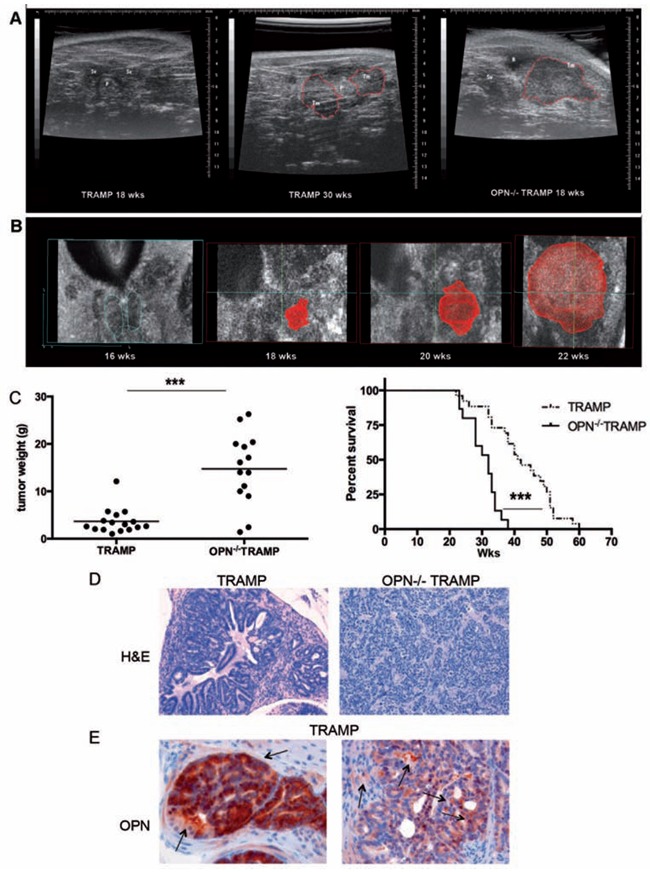
OPN^−/−^ TRAMP mice develop anaplastic tumors with increased frequency **A.** TRAMP and OPN^−/−^ TRAMP mice (*n* = 16 and 14, respectively) were followed for tumor growth by echographic ultrasound examination. Representative echographic abdominal imaging of prostate of 18wks (left) and 30wks old TRAMP (center) or 18 wks old OPN^−/−^TRAMP (right) mice are reported. Red dashed lines outline tumor mass. Scale bars at the borders are in millimeters. **B.** 3D rendering of echographic images of prostatic tumor growth (outlined in red) in a OPN^−/−^TRAMP mouse at 16, 20, 22 wks. **C.** Left: Mice followed echographically as described in (a) were euthanized at 30 wks of age. Graph reports weight of genitourinary apparatus of TRAMP (*n* = 16) and OPN^−/−^TRAMP (*n* = 14) as indicator of tumor burden (Student's *T* test: ***: *p* < 0.0001). Right: Alternatively, two other cohorts of TRAMP (*n* = 26) and OPN^−/−^ TRAMP (*n* = 15) mice were followed for survival; graph depicts Kaplan-Meier survival curve (Log Rank test: ***: *p* < 0.0001). **D.** Representative H&E staining of adenocarcinoma developing in 30 wks old TRAMP mice or anaplastic tumors grown in 18 wks old OPN^−/−^TRAMP mice. Magnification x200. At least 3 mice per group/age were analyzed and one representative picture is reported. **E.** Representative OPN staining in adenocarcinoma samples developing in 30 wks old TRAMP mice. Magnification x400. Arrows highlight positive cells. At least 3 mice per group/age were analyzed and one representative picture is reported.

We followed tumor onset and progression in OPN^−/−^TRAMP (*n* = 14) and TRAMP (*n* = 16) mice between 12 and 30 weeks (wks) of age by ultrasound (US) echographic examination. US allows evaluating prostate morphology and dimensions, and the elastomeric response to the echographic probe pressure, highlighting density alterations. The scan performed in 3D mode allows inspection of every prostatic lobe for the presence of tumor before it becomes palpable. US and histologic analyses of the prostate revealed no differences between the two strains until mice were 15 wks old. Between 18 and 22 wks of age a large, well-defined spheroid tumor appeared in OPN^−/−^TRAMP (Figure 1A and 1B) but not in age-matched TRAMP mice (Figure 1A). These spheroid tumors were even larger than the heterogeneous and multifocal tumors, also infiltrating the seminal vesicles, that characterize 30 wks old TRAMP mice (Figure 1A). Such growth differences were confirmed by weighting the genitourinary organs after necropsy at 30 wks of age or following mice survival (Figure 1C).

Histopathological analysis was performed on cohorts of mice sacrificed at different time points (18 to 20 wks, 20 to 30 wks, > 30 wks). Anaplastic lesions (Figure 1D, right) were present in roughly 60% of OPN^−/−^TRAMP mice in all cohorts, but only in few TRAMP mice and only after the 20th week of age (Table 1). Conversely, multifocal adenocarcinoma lesions (Figure 1D, left) were present in the majority of TRAMP mice at all time points (Table 1). As expected, positivity for OPN was found in both epithelial and stromal cells in TRAMP prostates (Figure 1E).

**Table 1:** Percentage of mutifocal or anaplastic lesions detected by histology in prostates of TRAMP or OPN−/− TRAMP mice at the indicated time points

	18<wks<20 (*n* = 7 TRAMP; *n* = 7 OPN−/−TRAMP)		20<wks<30 (*n* = 15 TRAMP; *n* = 15 OPN−/−TRAMP)		=30wks (*n* = 16 TRAMP; *n* = 14 OPN−/−TRAMP)	
	Multifocal lesions	Anaplastic lesions	Multifocal lesions	Anaplastic lesions	Multifocal lesions	Anaplastic lesions
**TRAMP**	100%	0%	78%	22%	93%	7%
**OPN −/− TRAMP**	50%	50%	40%	60%	46%	54%

**Figure 2:**
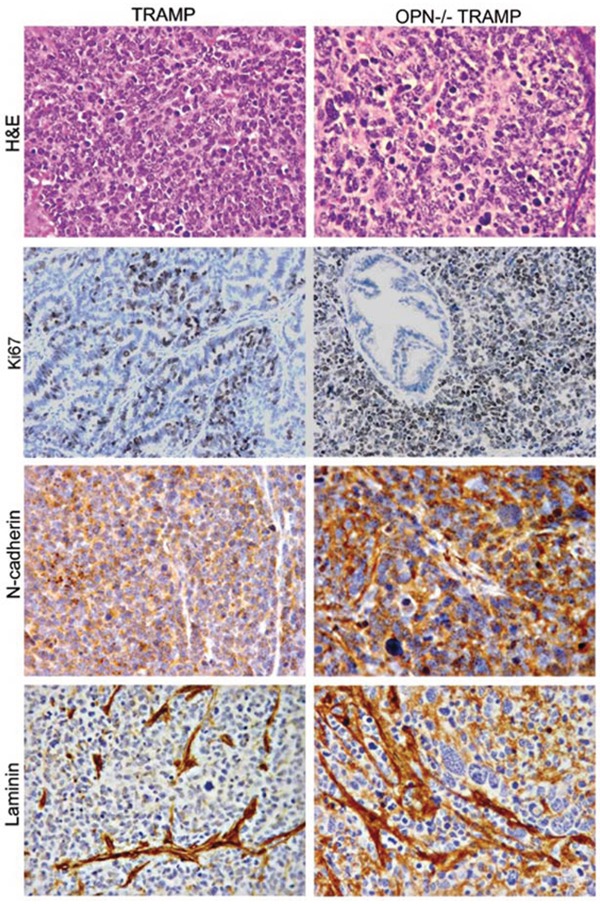
Phenotype of anaplastic lesions developing in TRAMP and OPN^−/−^TRAMP mice Representative hematoxilin and eosin (H&E) staining and immunohistochemistry for Ki67, N-cadherin and Laminin of anaplastic tumors developing in 30 wks old TRAMP and OPN^−/−^TRAMP mice as indicated. Magnification ×400. At least 3 mice per group were analyzed and one representative picture is reported.

To compare the anaplastic lesions from the two strains, tumors were collected at 30 wks of age for histopathology and immunohistochemistry. At this age, both strains develop poorly differentiated lesions. However, tumors formed in OPN^−/−^TRAMP mice were characterized by marked anaplasia and higher expression of Ki67, N-cadherin and laminin (Figure 2).

These data suggest that the lack of OPN at the beginning of the transformation process skews the nascent tumor towards a more aggressive, undifferentiated phenotype.

### Absence of OPN promotes androgen-independent tumor growth in the early stages of prostate carcinogenesis

**Figure 3:**
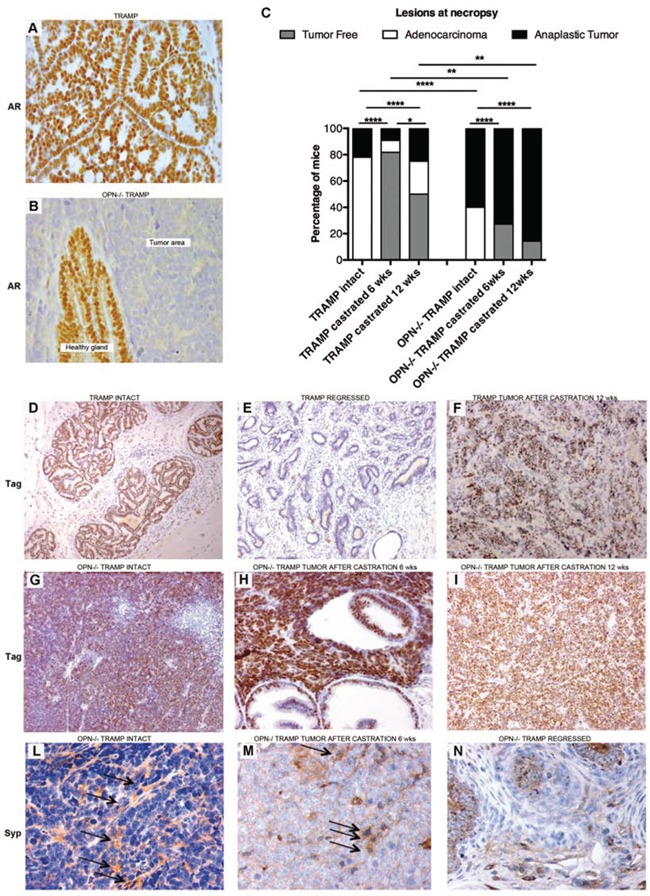
Anaplastic tumors developing in OPN^−/−^ TRAMP mice are androgen independent and express NE markers **A–B.** Representative immunohistochemistry staining of androgen receptor (AR) showing positivity in transformed prostates of 18 wks TRAMP mice (A), and in healthy glands surrounding tumor mass in age-matched OPN^−/−^TRAMP mice (B). Tumor areas in OPN^−/−^ TRAMP mice were AR negative (B). Magnification x400. At least 3 mice per group were analyzed and one representative picture is reported. **C.** Frequency of prostatic lesions in TRAMP and OPN^−/−^TRAMP either intact, or castrated at 6 (*n* = 11 for both groups) or 12 wks (*n* = 4 and 7, respectively) and sacrificed at 30 wks of age. (Fisher exact test *: *p* < 0.05; **: *p* < 0.01; ****: *p* < 0.0001) **D–F.** Representative Tag staining in prostate of intact TRAMP mice (D), in regressed prostate (E) or in anaplastic tumors (F) emerged in TRAMP mice after being castrated at 12 wks. Magnification × 200. **G–I.** Representative Tag staining in anaplastic prostate tumors developed in OPN^−/−^TRAMP mice either intact (G), castrated at 6 wks (H), or at 12 wks (I). **L–N.** Representative synaptophisin (Syp) staining in tumors developed in OPN^−/−^TRAMP mice either intact (L) or castrated at 6 wks (M) or in OPN^−/−^TRAMP mice showing prostate involution after castration (N). Arrows indicate positive cells. Magnification × 200.

Androgen receptor was expressed by normal and transformed glandular prostatic tissue of TRAMP mice (Figure 3A) as well as by normal epithelial cells of OPN^−/−^ TRAMP prostates, but not by tumors of OPN^−/−^ TRAMP mice (Figure 3B). This finding suggested that in OPN^−/−^TRAMP mice tumors could rise as androgen independent.

Androgen ablation is a standard treatment for patients with advanced PCa (www.cancer.gov/types/prostate/hp/prostate-treatment-pdq#section/all). Unfortunately these patients frequently develop androgen resistance and ineffective treatment options remain. This setting can be modeled in TRAMP mice by castration [13]. We investigated whether the lack of OPN affects mice susceptibility to hormone withdrawal in early phases of tumor development. TRAMP and OPN^−/−^TRAMP mice (both *n* = 11) were castrated before sexual maturation (6 wks) and tumor growth was monitored by US examination. Among castrated TRAMP mice, 9 showed prostate involution, 1 developed adenocarcinoma and the remaining one developed an anaplastic spheroid tumor with incidence similar to that of non-castrated TRAMP mice (12%; Figure 3C). Castrated OPN^−/−^TRAMP mice did not develop any evident adenocarcinoma, whereas 8 showed anaplastic tumors (73%, versus 60% of non-castrated littermates). Prostate involution occurred in the remaining 3 (27%) castrated mice (Figure 3C). Castration after sexual maturation (12 wks) did not change the incidence of anaplastic tumors in OPN^−/−^TRAMP mice, but increased up to 25% the rate of anaplastic tumors in TRAMP mice (Figure 3C) as expected [13, 14].

In TRAMP mice the expression of Tag is controlled by the rat Probasin promoter [6] which is regulated by androgens [15]. That means that Tag expression in TRAMP mice reflects androgen responsiveness of epithelial prostate cells. Immunohistochemistry revealed Tag expression by luminal cells of intact tumorigenic prostate glands of TRAMP mice (Figure 3D) but not by cells of involute prostates (Figure 3D). As already seen by others [13, 16], Tag expression was maintained in cells of anaplastic lesions occurring in castrated TRAMP mice (Figure 3F). Interestingly, Tag was expressed at high levels on anaplastic tumors of OPN^−/−^TRAMP mice regardless castration (Figure 3G–3I). These results confirmed that the anaplastic tumors arising in OPN^−/−^TRAMP mice developed as androgen-independent.

Synaptophysin staining confirmed the NE phenotype of anaplastic tumors [12] developed in both non-castrated (Figure 3L) and castrated (Figure 3M) OPN^−/−^TRAMP mice. Synaptophysin did not stain the epithelia of OPN^−/−^TRAMP prostates regressed because of castration (Figure 3N). Therefore, thereafter in this manuscript tumors from OPN^−/−^ TRAMP mice will be referred as NE tumors.

To test the possible OPN source either epithelial or hematopoietic that is relevant in this experimental model, bone marrow (BM) transplantation was performed to obtain chimeras lacking or not OPN in the prostatic epithelium or in the radio-resistant stroma cell components. In detail, BM from OPN-competent wild-type (WT) mice was transplanted into 6 wks old OPN^−/−^TRAMP mice as well as BM, either from OPN^−/−^ or WT mice, was transplanted into TRAMP mice. Mice were then sacrificed at 30 wks of age for histopathologic analysis of prostates. NE tumor frequency remained determined by the host genotype regardless that of donor BM, with high incidence in OPN^−/−^TRAMP and low incidence in TRAMP recipient chimeras (Figure S1). These results suggest that OPN produced by the host radio-resistant cell component contrast the occurrence of NE tumors.

### Basal origin of tumors in OPN^−/−^TRAMP mice

Whether prostatic NE cancers have a distinct cellular origin from adenocarcinomas or rather they originate from the peculiar differentiation of common precursors is still debated. As far as the TRAMP model is concerned, it is believed that NE tumors can arise from a subset of stem/progenitor cells populating the basal cell layer of glandular ducts [12, 17, 18]. We thus tested the expression of the basal p63, [19–21] and the luminal CK8 [22] markers in association with the cell proliferation marker Ki67.

**Figure 4:**
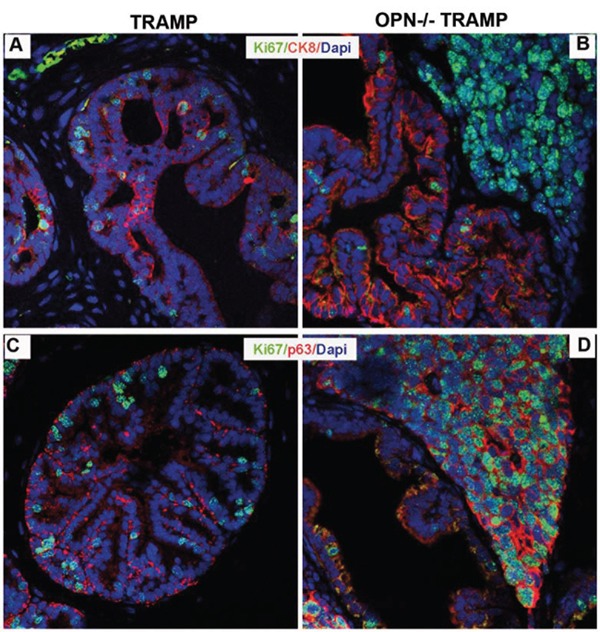
NE tumors of OPN^−/−^TRAMP mice have a basal phenotype **A–D.** Representative immunofluorescence staining of Ki67 (green) and CK8 (red; panels A and B) and of Ki67 (green) and p63 (red; panels C and D) in prostates of TRAMP and OPN^−/−^TRAMP mice of 15 wks of age. Blue: dapi. White arrows indicate cells co-expressing Ki67 and CK8, while yellow arrows indicate cells co-expressing Ki67 and p63. Three mice per group were analyzed and one representative picture is reported.

In prostate lobes of 15 wks old TRAMP mice, clusters of proliferating cells co-expressed Ki67 and CK8 (Figure 4A) whereas p63 positive cells were Ki67 negative (Figure 4C). On the contrary, in prostates of OPN^−/−^TRAMP mice luminal CK8 cells mostly lacked Ki-67 (Figure 4B), which instead co-localized with p63 in the basal compartment (Figure 4D). Notably, according to previous reports on prostate cancer patients [21], some cytoplasmic staining of p63 was detectable (Figure 4C and 4D) whereas nuclear p63 localization was canonic in normal prostate of wild-type mice (Figure S2).

### Gene expression profile confirms the NE phenotype of OPN^−/−^TRAMP tumors

To obtain a broad view of the transcriptional features distinctive of NE and adenocarcinoma lesions, we compared gene expression profiles of prostate samples collected from 18 weeks old OPN^−/−^TRAMP mice bearing macroscopic NE lesions (OPT-tm) with those from 30 wks old TRAMP mice (T30) representative of adenocarcinomas (ADC). A detailed description of samples used from microarray comparison is reported in Suppl. Table 1, and a detailed flow of samples comparison is shown in Suppl. Figure 3

**Figure 5:**
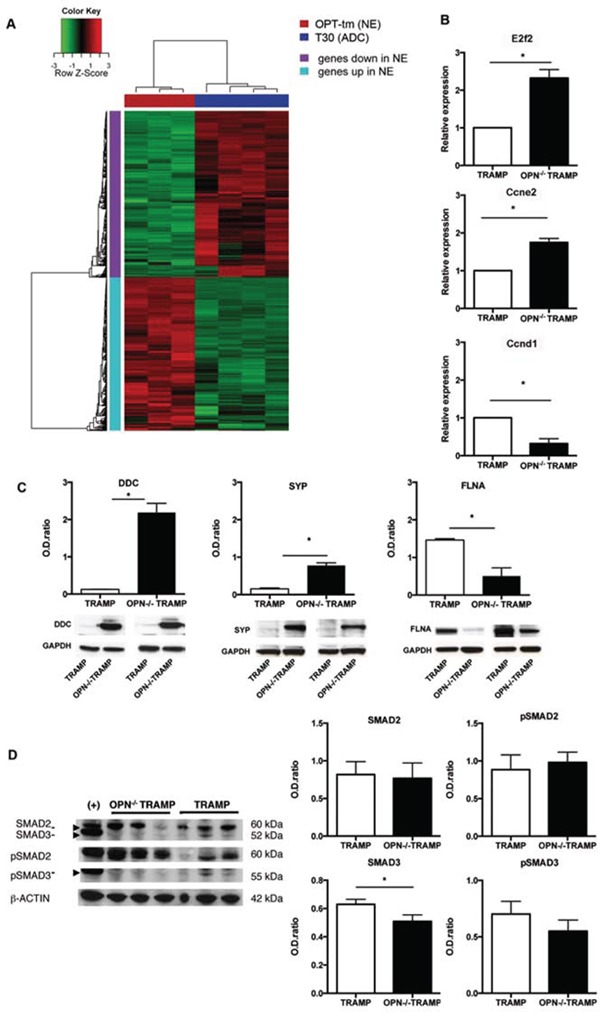
Gene expression profile confirms NE phenotype of OPN^−/−^TRAMP tumors **A.** Heatmap of differentially expressed genes in NE tumors of 18 wks old OPN^−/−^TRAMP (OPT18_tm) vs adenocarcinoma (ADC) of 30 wks old TRAMP (T30) mice. Red and green color indicates relative expression as indicated. Refer to Suppl. Table 1 and Suppl. Figure 3 for detailed description of samples analyzed and flow chart of comparisons. **B.** Relative expression +/− SD of E2f2, Ccne2 and Ccnd1 in prostates of 30 wks TRAMP and 18 wks OPN−/−TRAMP mice as assessed by real-time PCR. Experiment was repeated twice with samples from three different mice per group. **C.** Western blot analysis for L-Dopa Decarboxylhase (DDC), synaptophysin (SYP) and Filamin (FLNA) in prostates of 30 wks TRAMP and 18 wks OPN−/−TRAMP mice. Values are quantified to internal control (GAPDH). Picture reports cropped blots around the specific bands. Experiment was repeated twice with samples from two different mice per group. **D.** Western blot analysis for SMAD2, SMAD3 and their phosphorylated forms (pSMAD2 and pSMAD3, respectively) or β-actin (as control) in prostates of 30 wks TRAMP and 18 wks OPN−/−TRAMP mice. HeLa cells stimulated with TGFb were used as positive control (+). Values are quantified to internal control (β-actin). Picture reports cropped blots around the specific bands Experiment was repeated twice with samples from three different mice per group. Student's *T* test: *: *p* < 0.0001.

Unsupervised hierarchical clustering clearly distinguished gene expression profiles of TRAMP mice with adenocarcinoma from OPN^−/−^TRAMP mice with NE tumor (Figure S4). We found 2536 differentially expressed genes between the two classes (absolute fold change ≥ 2 and FDR < 5%; Heatmap in Figure 5A). To identify biological processes distinguishing the two histotypes we performed Gene Set Enrichment Analysis (GSEA) using gene sets compiled from canonical pathway and Gene Ontology databases. Significant gene sets (FDR < 5%, *p*-value < 0.001) were visualized as interaction networks using the Enrichment map plugin in Cytoscape (Figure S5). NE tumors were characterized by the activation of gene sets involved in cell cycle and proliferation, transcription and RNA-processing, and neural system. These findings were confirmed by functional analysis of the differentially expressed genes using Ingenuity Pathway Analysis (IPA) software. In OPT-tm, among genes involved in cell cycle, *E2f2* and *Ccne2* were the most up-regulated, while *Ccnd1*, whose up-regulation has been reported in TRAMP mice [23, 24], was the most down-regulated. These results were confirmed by RT-qPCR analysis (Figure 5B) and were concordant with the high cellular proliferative rate observed both macroscopically and histologically in OPN^−/−^TRAMP tumors. The most up-regulated genes in OPT-tm samples were those involved in neurological diseases and in neuronal system development, including L-Dopa Decarboxylase (*Ddc*, fold change = 172.2) and synaptophisin (*Syp*, fold change = 31.3), confirming the NE phenotype of anaplastic tumors arising in OPN^−/−^TRAMP mice. Upregulation at protein level of DDC and SYP in NE tumors was validated by western blot (Figure 5C).

IPA predicted *Tgfb1* to be an uprstream regulator of the differentially expressed genes in OPT-tm samples (*p* = 8.93e-37). Indeed 157 targets of *Tgfb1* were listed as differentially expressed genes and 84 of them had an expression consistent with Tgfb1 pathway inhibition. This finding was supported by the significant down-regulation of *Tgfb*1 in OPT-tm (FDR=0.005), although this gene was excluded from the list of differentially expressed genes because of a fold-change of −1.53. Moreover, Filamin A (FLNA), a key TGFβ activator [25], was among the down-regulated genes in our signature and western blot confirmed FLNA protein down regulation (Figure 5C).

*Tgfbr2* and *Tgfbr3* transcripts were significantly down-regulated in OPT-tm, whereas *Tgfbr1*, which is responsible for SMADs phosphorylation and activation, was up-regulated. As protein levels of TGFbRI and TGFbRII were comparable between OPN^−/−^TRAMP NE tumors and TRAMP adenocarcinoma (not shown), we focused on proteins downstream the TGFβ pathway, particularly Smad2 and Smad3, which are crucial mediators of TGFβ activity in PCa [26]. *Smad3* was down-regulated in GEP and in western blot in NE tumors from OPN^−/−^TRAMP mice (Figure 5D). On the contrary, *Smad2* transcript and protein, as well as phosphorylation of SMAD3 and SMAD, remained unchanged in OPN^−/−^TRAMP NE tumors versus TRAMP adenocarcinomas (Figure 5D). These results suggested that in absence of OPN the TGFβ pathway and the cell cycle were down-regulated, likely through the activity of FLNA and SMAD3.

### NE signature from early lesions of OPN^−/−^ TRAMP mice is enriched in genes overexpressed in human NE prostate and can predict NE tumor outcome

**Figure 6:**
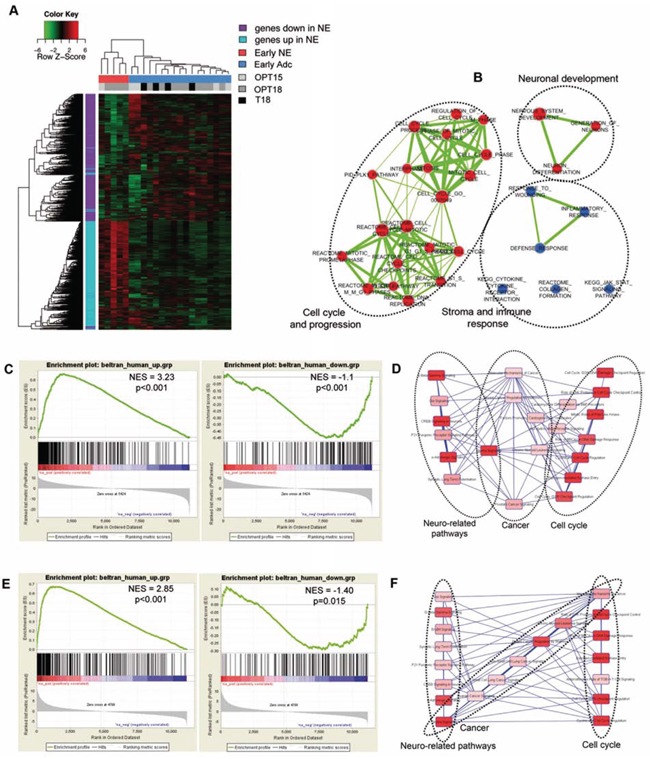
Early NE signature from OPN^−/−^TRAMP mice correlates with human NE tumors **A.** Hierarchical clustering of early samples obtained from mice free of detectable prostatic lesions (OPT^−/−^TRAMP 18 wks: light grey; OPN^−/−^TRAMP 15 wks: dark grey and TRAMP 18 wks: black). Refer to Suppl. Table 1 and Suppl. Figure 3 for detailed description of samples analyzed and flow chart of comparisons. **B.** Enrichment map showing gene sets significantly enriched in *Early-NE* compared to *Early-ADC* according to GSEA. Nodes represent gene sets. Node size is proportional to the number of genes in the gene set. Edges connect overlapping gene sets. Edge thickness indicates the size of the overlap. Red indicates up-regulation, blue indicates down-regulation. **C.** GSEA plot showing enrichment of genes up-regulated or down-regulated in human NE tumors towards genes over-expressed (left) or down-regulated (right) in OPT-tm compared to T30 mice. **D.** Canonical pathways, identified by functional analysis with IPA of the core set of genes from human NE samples leading the enrichment in NE OPT-tm samples. **E.** GSEA plot showing enrichment of genes up-regulated or down-regulated in human NE tumors towards genes over-expressed (left) or down-regulated (right) in *Early-NE* compared to *Early-ADC* samples. **F.** Canonical pathways, identified by functional analysis with IPA of the core set of genes from human NE samples leading the enrichment in *Early-NE* samples.

We have the idea to test the possibility of defining a gene signature that could predict the onset of NE disease in PCa patients. Using the 2536 genes distinguishing NE from adenocarcinoma prostate tumors, we applied unsupervised hierarchical clustering to gene expression data from prostates of TRAMP mice of 18 wks of age (T18, *n* = 4) and from OPN^−/−^ TRAMP mice of 15 (*n* = 5) or 18 (*n* = 9) wks of age (OPT15 and OPT18, respectively) all devoid of US-detectable lesions (Table S1 and Figure S3 describe samples and class comparisons). We found that early prostate samples fell into two distinct clusters (Figure 6a). The first cluster was composed of 5 samples, hereafter called *Early-NE*, whose gene expression pattern resembled that of NE OPT-tm. These samples were all from OPN^−/−^TRAMP mice (4 of 18 wks old and 1 of 15 wks), indicating that at the time of sacrifice they were likely bearing a developing NE tumor, well before any possible diagnosis. The second cluster contained 17 samples, hereafter called *Early-ADC*, whose gene expression profile was similar to that of TRAMP adenocarcinomas. All early samples from TRAMP (T18) and from OPN^−/−^ TRAMP mice, without NE features, fell into this cluster. To identify early markers specific for NE tumor a comparison between *Early-NE* and *Early-ADC* GEPs was performed. We found that all the 184 differentially expressed genes (absolute fold change ≥ 2 and FDR < 5%), were upregulated in the *Early-NE* group (Figure 6A). According to GSEA these genes are involved in cell cycle and nervous system development, but also in down-regulation of pro-inflammatory processes (Figure 6B, Table S2 and S3). This list of 184 genes provides a transcriptional profile distinguishing NE tumors from adenocarcinoma at early stages of transformation.

We were interested in asking whether the transcriptional alterations observed in our mouse models could find correlation with human tumors counterpart. As gene expression datasets containing both NE and adenocarcinoma human PCa are not publicly available we used GSEA to test whether genes previously identified as up- or down- regulated in human prostate NE tumors versus prostate adenocarcinomas [27] were positively or negatively enriched in mouse NE lesions. Results showed that genes over-expressed in NE human tumors were also positively enriched in both OPT-tm and *Early-NE* samples (Figure 6C, left, and Figure 6E, left, respectively). The core enrichment genes, accounting for the gene set's enrichment, were related to cancer, neuronal development, cell growth and cell cycle progression (Figure 6D and 6F, and Table S4 and S5), confirming the results obtained with the murine NE signatures. Conversely, genes down-regulated in NE patients were significantly negatively enriched in both OPT-tm and *Early-NE* samples while up-regulated in T30 (late adenocarcinoma) and *Early-ADC* samples (Figure 6C, right, and Figure 6E, right, respectively). Taken together these results showed that gene expression profiles of tumors arising in OPN^−/−^TRAMP mice were similar to those of human NE prostatic cancers.

## DISCUSSION

ECM confers proper architecture and function to tissues. It regulates connections and communication between cells and is responsible for the continued tissue remodeling in development or healing. Of great interest is the role of ECM in cancer, representing a tissue that does not heal [28], especially in premalignant to malignant transition [29].

OPN is an ECM non-structural protein, member of the SIBLINGs, endowed with pro-migratory and pro-angiogenic properties in transformed tissues [30] and considered a potential tumor marker [31]. In PCa its expression in primary tumors [4] and bone metastases [32] correlates with poor prognosis and its targeting has been considered a therapeutic option [33].

Despite the numerous studies, none has investigated the role of OPN at onset of PCa. The present work is the first to study the effect of OPN genetic deficiency in TRAMP, a mouse model mimicking human PCa. Considering its high expression in advanced human PCa we would have expected that OPN knockout in TRAMP mice would have delayed tumor onset or reduced tumor size. To our surprise, OPN^−/−^TRAMP mice displayed early onset and accelerated tumor development in comparison to TRAMP mice. These tumors developed as spherical masses, macroscopically detectable at 18 wks of age by echographic examination. Histopathology classified these tumors as undifferentiated and immunohistochemistry indicated the loss of AR, the retention of Tag and the gaining of synaptophysin. The same characteristics have also been described for NE tumors that seldom develop spontaneously in TRAMP mice unless stimulated by castration [12, 18]. Castration in TRAMP mice has different outcomes depending on the age of mice at surgery. Before sexual maturation it causes prostate involution and prevents neoplastic transformation [13] whereas at late stages it promotes the development of androgen independent NE tumors; which express high levels of Tag [13, 16, 34]. In OPN^−/−^TRAMP mice castration at either 6 or 12 weeks had no effect on NE tumor development, which still occurred at high rate in an hormone independent way.

We also found that proliferating Ki67 positive cells in prostates of OPN^−/−^TRAMP mice express cytoplasmic p63, thus confirming that the basal prostate compartment is transforming in OPN^−/−^TRAMP mice. This is in line with the hypothesis that NE tumors arise from basal stem-like cells in the TRAMP model [12]. The cytoplasmic staining of p63 (Figure 4D) is in agreement with human PCa, carrying a translocation of p63 from the nucleus to the cytoplasm, an event that has been correlated with increased cell proliferation and dismal prognosis [21].

Clustering analysis performed on gene lists originated by prostate samples from OPN^−/−^ TRAMP of 18 wks with US detectable NE tumors and by samples from 30 wks TRAMP with adenocarcinoma, proved the distinct nature of tumors developing early in mice deficient for OPN.

*In silico* analysis showed that genes promoting cell cycle were most up-regulated in OPN^−/−^TRAMP than TRAMP tumors. Among them CyclinE2 and E2f2 are inducible by estrogens [35], indicating their possible involvement in the development of androgen independent NE tumor [36]. CyclinD1, which promotes cell cycle progression in TRAMP adenocarcinomas [37–39], was down-regulated in NE tumors from OPN^−/−^TRAMP mice. These data are in line with the observation that up to 90% of human NE xenografts show loss of Cyclin D1 [40]. The similarity between murine and human NE tumors was corroborated by the concordant up-regulation of DDC, a biomarker for human NE PCa [41, 42].

The key genes down-regulated in our signature belong to the TGFβ pathway and encode for cytoskeletal proteins. TGFβ mediates the activity of OPN in maintaining hematopoietic stem cells quiescence in the mesenchymal BM niche [43]. Not surprisingly, it might also maintain dormant the prostatic stem cell compartment [26]. The lack of OPN in the prostate stem cell niche may inactivate the TGFβ pathway and therefore favor the escape from its anti-proliferative control, towards NE tumors development.

In keratinocytes Filamin A (FLNA), a cytoskeletal SMAD-binding protein, binds SMAD2 for effective TFGβ receptor signaling; accordingly, FLNA-deficient melanoma cells have impaired TGFβ signaling [25]. In OPN^−/−^TRAMP NE tumors, FLNA was down-regulated at gene and protein levels. SMAD3 gene and protein were also down-regulated although the residual protein was still phosphorylated. SMAD3 is a key intermediated in the TGFβ pathway [44] and its depletion cannot be overcome by SMAD2 in sustaining TGFβ–mediated cell cycle arrest and growth inhibition [45]. Accordingly, a reduced expression of SMAD3 diminishes the tumor-suppressor function of the TGFβ pathway in a model of acute T-cell lymphoblastic leukemia [46]. Overall, our data support the conclusion that deregulation of TGFβ signaling is responsible for increased tumor proliferation and aggressiveness in the OPN^−/−^ TRAMP model.

Nested class comparison between *Early-NE* vs *Early-ADC* tissues identified 184 genes that were all up-regulated in prostates from OPN−/−TRAMP at the onset of the still undetectable NE tumors, and that might contain possible biomarkers for early diagnosis.

The validation of OPN^−/−^TRAMP as a reliable model for human NE prostate cancer came from the GSEA analysis on Beltran dataset of PCa patients [27]. Indeed, genes that are up-regulated in human NE prostate cancers were also enriched in our data set from OPN^−/−^TRAMP mice. Common were the genes belonging to canonical pathway related to cancer, neuronal development, cell growth and cell cycle progression.

Modification of the genetic background has been found to change the frequency of NE tumors in TRAMP mice [47], suggesting that polymorphic germline modifier genes may favor NE tumors in this model. Here, using the same C57BL/6 genetic background, we confined to OPN the genetic difference, underscoring a role of OPN in controlling prostate NE tumor outcome. Although this study is the first to demonstrate the role of OPN in prostate tumorigenesis, another SIBLINGs member, namely SPARC, has been shown to favor the occurrence of more aggressive cancers, of undefined histotype in TRAMP mice, if deleted [48]. Altogether these data support the idea that under the same oncogenic driver (SV40 large T antigen), the ECM composition can influence the phenotype and the aggressiveness of the upcoming tumor. Conversely, in advanced PCa patients both OPN and SPARC overexpression are associated with poor prognosis and metastatic phenotype [4, 32, 49], implying that the same ECM proteins can exert different functions at different tumor stages.

## MATERIALS AND METHODS

### Mice

TRAMP mice (C57BL/6-tgN (TRAMP)8247 Ng) were kindly provided by Dr. Vincenzo Bronte (IRCCS Istituto Oncologico Veneto, Padova, Italy), under agreement with Dr. Norman Michael Greenberg (Fred Hutchinson Cancer Research Center, Seattle, USA). TRAMP mice were maintained heterozygous (TRAMP^+/−^) and screened according to [6]. OPN knockout mutant B6.129S6(Cg)-*Spp1^tm1Blh^*/J (OPN^−/−^) mice were purchased from Jackson Laboratories and intercrossed to TRAMP mice, in order to obtain congenic B6.tgN (TRAMP)8247 Ng Spp1<tm1Blh>/J (OPN^−/−^TRAMP). Female mice heterozygous for Tag (TRAMP^+/−^) and OPN^−/−^ were bred with male OPN^−/−^ mice to obtain experimental OPN^−/−^TRAMP^+/−^ mice, which were genotyped for Tag expression [6]. Mice were maintained under pathogen-free conditions at the animal facility of Fondazione IRCCS Istituto Nazionale dei Tumori. Animal experiments were authorized by the Institute Ethical Committee and performed in accordance to institutional guidelines and national law (D.lgs 26/2014). Prostate tumor growth was echo-graphically monitored using a Vevo 770 micro-ultrasound imaging system (Visualsonics Inc., Toronto, Canada) as described [50]. Castration was performed after anesthesia (100 mg/kg Ketamine, 16 mg/kg Xylazine) as described [51].

### Histology, immunohistochemistry

Prostates were fixed in formalin and embedded in paraffin. 5 μM sections were stained with Mayer-Hematoxylin and Eosin (H&E). Alternatively, after re-hydratation and antigen retrieval, immunohistochemistry was performed using the streptavidin-biotin-peroxidase complex method, and 3,3′-Diaminobenzidine tetrahydrochloride as chromogenic substrate. Primary antibodies were: Ki67 (TEC-3, Dako, Glostrup Denmark), N-cadherin (Abcam, Cambridge, MA, USA), Laminin (Biodesign, Memphis, TN, USA), Androgen Receptor (Merck Millipore, Milano, Italy) Synaptophysin (Abcam,), SV40 Tag (clone pAb101, BD Biosciences, Buccinasco Italy), OPN (AbCam). Slides were analyzed under a Leica DM2000 optical microscope equipped with a Leica DFC320 digital camera (Leica Microsystems, Milan, Italy).

### Immunofluorescence

Sections were incubated with primary antibody anti Ki67 o/n at 4°C, then washed in PBS and incubated for 1h with an Alexa-488 anti-rabbit antibody. Slides were then incubated with anti CK8 or anti p63 antibodies (Abcam) for 1h, washed and further incubated with Alexa-568 anti-mouse antibody. Sections were counter stained with DAPI and analyzed with a Microradiance 2000 (Bio-Rad Laboratories, Segrate, Italy) confocal microscope equipped with Ar (488 nm), HeNe (543 nm) and red laser diode (638 nm) lasers. Confocal images (512 × 512 pixels) were obtained using a x200, 0.5 NA Plan Fluor DIC or x600 1.4 NA oil immersion lens and analyzed using ImagePro 7.0.1 software.

### RT-qPCR

Prostates were lysed with TRIzol (Invitrogen, Life Technologies, Monza, Italy) and RNA was purified by phenol/chloroform extraction followed by loading onto RNeasy MINI kit (Qiagen, Milan, Italy). On-column DNAse treatment was performed. RNA purity and yield was assessed using NanoDrop ND-100 Spectrophotometer (NanoDrop Technologies, Wilmington, DE).

RNA was Reverse Transcribed using High Capacity cDNA Reverse Transcription Kit (Applied Biosystems). RT-qPCR reaction was prepared using TaqMan (R) Fast Universal PCR Master Mix and run on a 7900 HT Fast Real-time PCR System (Applied Biosystems, Life Technologies, Monza, Italy) as described previously [52]. The following TaqMan(R) probes were used: *Gapdh* (Mm99999915_g1), *Ccne2* (Mm00438077_m1), *Ccnd1* (Mm00432359_m1), E2f2 (Mm00624964_m1), *Ddc* (Mm00516688_m1), *Syp* (Mm00436850_m1). Expression values were normalized to internal control (Gapdh) and to control sample (TRAMP prostate) calculating the ΔΔCT.

### Western blot

Ten sections of 10 mm from frozen samples of TRAMP or OPN^−/−^TRAMP tumors were lysed, run on a 4-12% Bis-Tris Gel (Invitrogen) and transferred on a NC membrane (Amersham, GE Healthcare, Milano, Italy). Primary antibodies against Smad3, Smad2, phospho Smad2, phospho Smad3, Filamin, DDC and Syp were from Cell Signaling Technology, against β-actin from SIGMA Aldrich. Secondary HRP antibodies were goat anti-rabbit and rabbit anti-goat (Invitrogen). Densitometric analysis was performed using the ImageJ 1.48v software (http://imagej.nih.gov/ij).

### Gene expression profiling

We collected prostates from 18 wks old TRAMP mice (T18, *n* = 4), with null or minimal disease, from 30 wks old TRAMP mice (T30, *n* = 4) in which adenocarcinoma was palpable and detectable by US, from OPN^−/−^TRAMP mice of 15 or 18 wks of age (OPT15, *n* = 5 and OPT18, *n* = 9, respectively) without any detectable lesion and from 18 wks old OPN^−/−^TRAMP mice with detectable NE tumor mass (OPT18tm, *n* = 3). A detailed description of samples used for microarray comparison is in Suppl. Table 1, and a detailed flow chart depicting how samples were compared is in Suppl. Figure 3

RNA was isolated using the guanidinium thiocyanate/cesium chloride gradient method [53]. RNA concentrations were measured with the NanoDrop ND-100 Spectrophotometer (NanoDrop Technologies) while RNA quality was assessed with the Agilent 2100 Bioanalyzer (Agilent Technologies, Palo Alto, CA, USA) using the RNA 6000 Nano kit (Agilent Technologies). The mean RIN value was 8.028 (SD = 0.65). RNA samples were processed for microarray hybridization by the Functional Genomics core facility at Fondazione IRCCS Istituto Nazionale dei Tumori, Milan. Briefly, 800 ng of total RNA was reverse transcribed, labeled with biotin and amplified overnight (14 hours) using the Illumina RNA TotalPrep Amplification kit (Ambion, Life Technologies, Grand Island, NY, USA) according to manufacturer's protocol. One μg of the biotinylated cRNA sample was mixed with the Hyb E1 hybridizatioin buffer containing 37.5% (w/w) formamide and then hybridized to array MouseRef-8 v2.0 Expression BeadChip (Illumina, Inc., San Diego, CA, USA) at 58°C overnight (18 hours). Array chips were washed with manufacturer's E1BC solution, stained with 1 μg/ml Cy3-streptavidine (Amersham Biosciences; GE Healthcare, Piscataway, NJ, USA) and scanned with Illumina BeadArray™ Reader.

### Microrarray data pre-processing

Raw expression data were collected from scanned images using Illumina BeadStudio v3.3.8 and processed using the *lumi* package from Bioconductor [54]. Quality control was performed on raw and processed data by evaluation of array intensity distributions, distances between arrays, and by principal component analysis for the identification of possible outliers. All samples passed quality-control procedures. Raw data were log_2_-transformed, normalized with robust spline normalization and filtered, keeping only the probes with a detection *p*-value < 0.01 in at least one sample. Among probes mapping on the same gene the most detected one was chosen. All array data have been deposited in NCBI's Gene Expression Omnibus and are accessible through GEO Series accession number GSE69903.

### Microarray data analysis

Differentially expressed genes between classes were identified using the *limma* package [55]. *P*-values were corrected for multiple testing using the Benjamini-Hochberg false discovery rate method. An FDR < 0.05 and an absolute fold change ≥ 2 were used as criteria for selection of differentially expressed genes. Agglomerative hierarchical clustering was performed using average linkage and 1-Pearson's correlation coefficient as distance measure. Gene Set Enrichment Analysis (GSEA [56]) was applied to identify biological processes enriched in the different groups. Gene sets were retrieved from the c2 canonical pathways and c5 biological process collections from the MSigDB database (http://www.broadinstitute.org/gsea/msigdb/index.jsp). Significantly enriched gene sets (*p* < 0.001 and FDR< 0.05) were visualized using Cytoscape v2.8.3 and the Enrichment Map plugin [57]. Functional analyses of gene lists were generated through the use of IPA(QIAGEN, www.qiagen.com/ingenuity).

### Statistical analysis

We used GraphPad Prism software (GraphPad Software, La Jolla, CA, USA). Student's *t*-test was used for comparison of quantitative variables. Association between categorical variables was carried out using Fisher's exact test. Log-Rank test was applied for survival curves. All statistical tests were two-sided and a *p*-value <0.05 was considered significant.

## SUPPLEMENTARY FIGURES AND TABLES


