# Supplementary material for: Genetic deletion of osteopontin in TRAMP mice skews prostate carcinogenesis from adenocarcinoma to aggressive human-like neuroendocrine cancers
Source: Oncotarget. 2015 Dec 19;7(4):3905–20. doi: 10.18632/oncotarget.6678 (PMC4826179; doi:10.18632/oncotarget.6678)
Supplement: Supplementary file 1 [file oncotarget-07-3905-s001.pdf]

SUPPLEMENTARY FIGURES AND TABLES

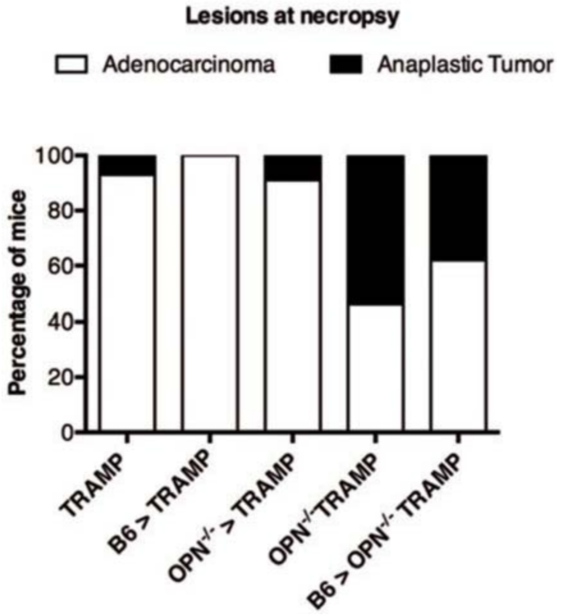

Supplementary Figure S1: OPN replacement in bone marrow derived cells does not change NE tumor frequency. Frequency of prostatic lesions in TRAMP and OPN$^{-/-}$TRAMP either untreated, or irradiated (1200 RAD) and transplanted with 10^6 bone marrow (BM) cells from C56BL6 (B6) or OPN-/- donors and sacrificed at 30 wks of age.

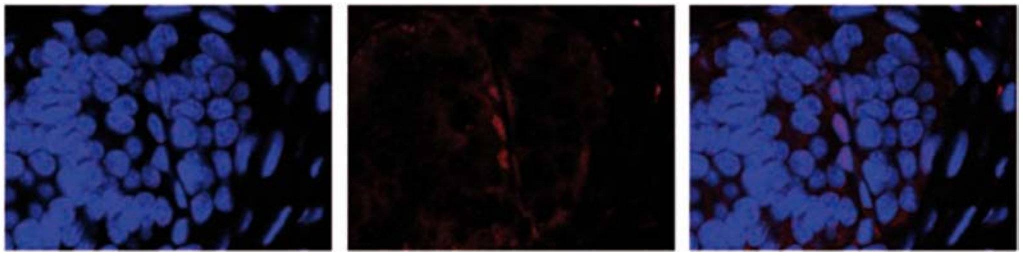

Supplementary Figure S2: Wild type prostates have nuclear p63. Representative immunofluorescence staining showing p63 (red) in prostate of C57BL/6 mice. Blue: dapi.

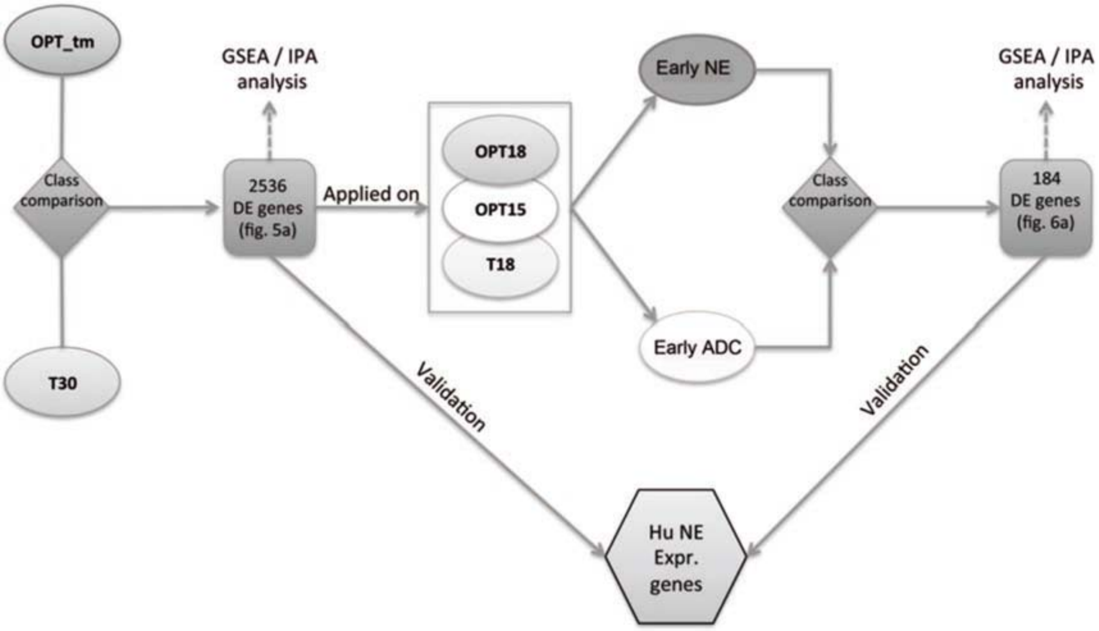

Supplementary Figure S3: Gene expression analysis. Flow chart of exploited Class Comparisons and subsequent analyses of microarray data performed on prostates samples of TRAMP and OPN$^{-/-}$TRAMP mice.

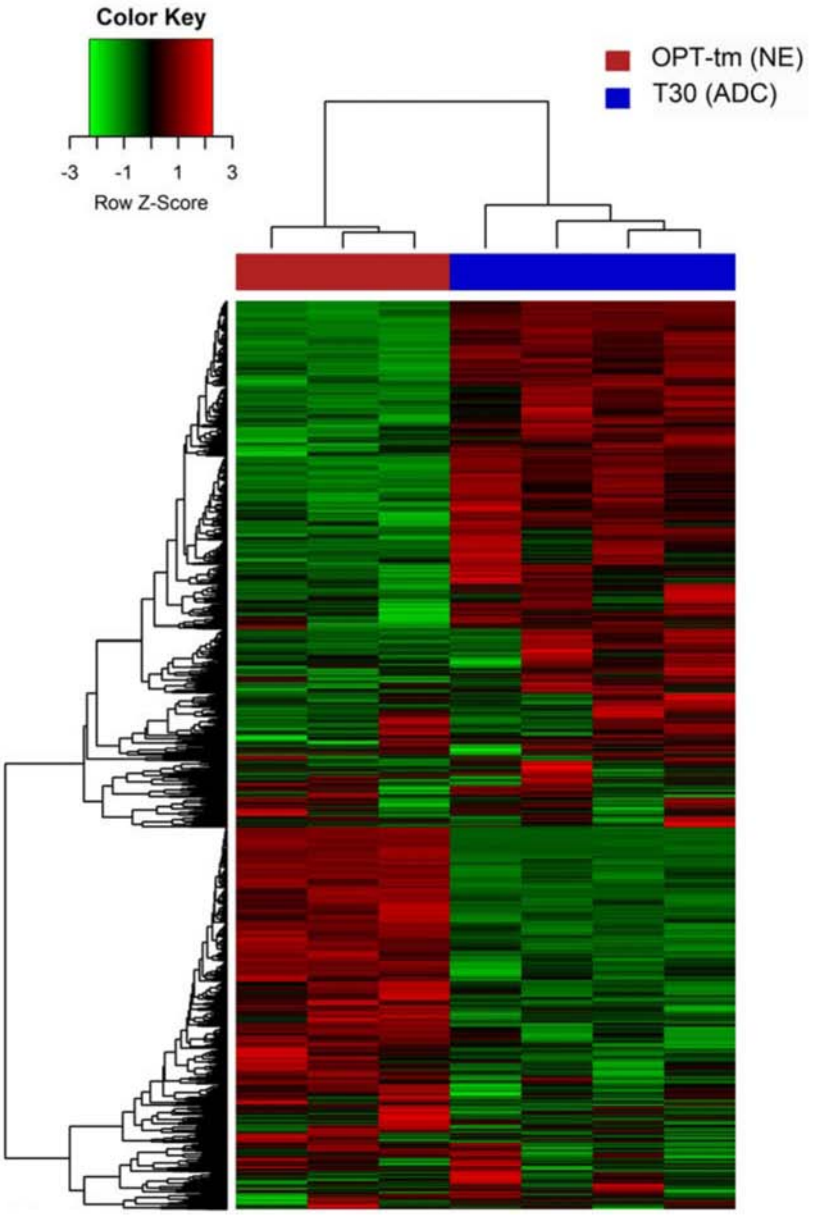

Supplementary Figure S4: Unsupervised hierarchical clustering of gene expression comparing TRAMP versus OPN$^{-/-}$ TRAMP tumors. Unsupervised hierarchical clustering of gene expression profiles obtained from adenocarcinoma (ADC) arising in TRAMP mice of 30 weeks of age (T30) versus NE tumors arising in OPN-/-TRAMP mice (OPT-tm), using the first 5000 most variable genes according to interquartile range. Dendrogram of samples shows a clear separation between mice with adenocarcinoma from those with a NE tumor.

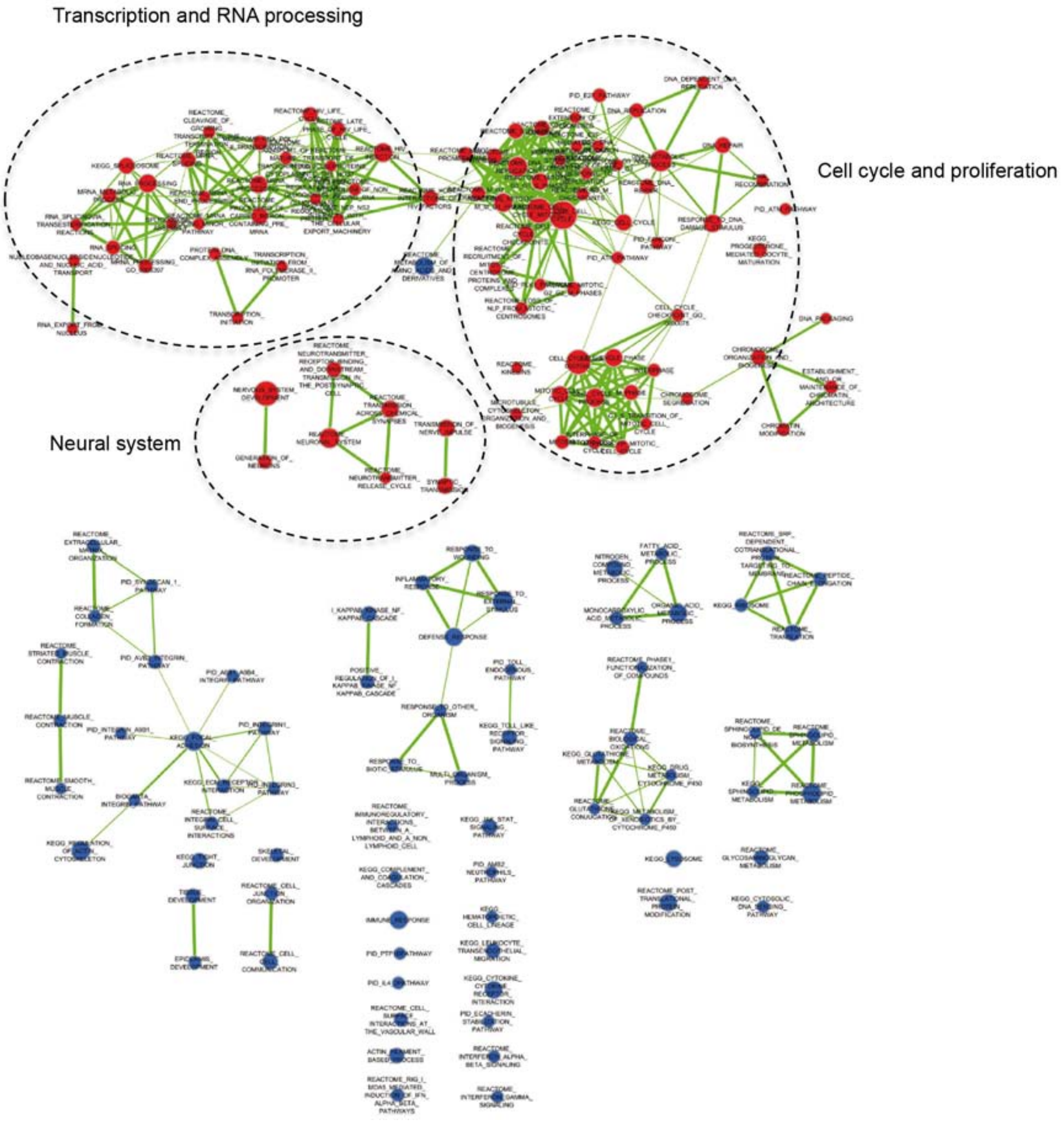

Supplementary Figure S5: Gene Set Enrichment Analysis of differentially expressed genes between NE and adenocarcinoma prostate tumors. Enrichment map showing gene sets differential expressed between OPT_tm (NE) and T30 (adenocarcinoma) samples. Nodes represent gene sets. Node size is proportional to the number of genes in the gene set. Edges connect overlapping gene sets. Edge thickness indicates the size of the overlap. Red indicates up-regulation, blue indicates down-regulation.

Supplementary Table S1: Prostates samples of TRAMP and OPN$^{-/-}$TRAMP mice used for microarray analysis

Chip_ref.	ID mouse	Nick	Genotype	Age (weeks)	Phenotype
X4700266073_F	OPT15_2956	OPT15	OPN-/- TRAMP	15	Undetectable tumor
X4700266115_F	OPT15_2957	OPT15	OPN-/- TRAMP	15	Undetectable tumor
X4700266084_F	OPT15_2962	OPT15	OPN-/- TRAMP	15	Undetectable tumor
X4700266109_E	OPT15_3020	OPT15	OPN-/- TRAMP	15	Undetectable tumor
X4700266109_F	OPT15_3022	OPT15	OPN-/- TRAMP	15	Undetectable tumor
X4700266073_A	OPT18_1327	OPT18	OPN-/- TRAMP	18	Undetectable tumor
X4700266073_B	OPT18_1328	OPT18	OPN-/- TRAMP	18	Undetectable tumor
X4700266073_C	OPT18_1329	OPT18	OPN-/- TRAMP	18	Undetectable tumor
X4700266073_D	OPT18_1330	OPT18	OPN-/- TRAMP	18	Undetectable tumor
X4700266115_B	OPT18_443	OPT18	OPN-/- TRAMP	18	Undetectable tumor
X4700266115_C	OPT18_1322	OPT18	OPN-/- TRAMP	18	Undetectable tumor
X4700266115_D	OPT18_450	OPT18	OPN-/- TRAMP	18	Undetectable tumor
X4700266115_E	OPT18_1339	OPT18	OPN-/- TRAMP	18	Undetectable tumor
X4700266084_A	OPT18_1325	OPT18	OPN-/- TRAMP	18	Undetectable tumor
X4700266084_B	OPT18_444	OPT18	OPN-/- TRAMP	18	Undetectable tumor
X4700266084_C	OPT18_2967	OPT18	OPN-/- TRAMP	18	Undetectable tumor
X4700266084_D	OPT18_1336	OPT18	OPN-/- TRAMP	18	Undetectable tumor
X4700266109_D	OPT18_1323	OPT18	OPN-/- TRAMP	18	Undetectable tumor
X4700266073_E	OPT18_1333_tm	OPTtm	OPN-/- TRAMP	18	NE
X4700266084_E	OPT18_1340_tm	OPTtm	OPN-/- TRAMP	18	NE
X4700266109_B	OPT18_1335_tm	OPTtm	OPN-/- TRAMP	18	NE
X4700266073_H	T18_1545	T18	TRAMP	18	Undetectable tumor
X4700266115_H	T18_421	T18	TRAMP	18	Undetectable tumor
X4700266084_H	T18_408	T18	TRAMP	18	Undetectable tumor
X4700266109_H	T18_1578	T18	TRAMP	18	Undetectable tumor
X4700266073_G	T30_6771	T30	TRAMP	30	Adenocarcinoma
X4700266115_G	T30_6768	T30	TRAMP	30	Adenocarcinoma
X4700266084_G	T30_6775	T30	TRAMP	30	Adenocarcinoma
X4700266109_G	T30_6769	T30	TRAMP	30	Adenocarcinoma

Supplementary Table S2: Genes up-regulated in Early NE samples: functional network analysis

Top Network	Score	Focus Protein	Molecules
Cellular Growth and Proliferation, Neurological Disease, Skeletal and Muscular Disorders	37	23	Alpha tubulin,**AMPH, BDNF, BIRC5, CCNB1, CCNE2, CD1D, CDK5R1,** Cg, **CHGA, CYP3A4, DPYSL2**,E2f,**E2F2**,ERK, **HEY1**, HEY2, HIST1H2AB,**KIF22**, Map4K4, **MAPT, MYBL2**, NFkB (complex),Notch,P38 MAPK, **PBK**, **PKIA,** PPP2R5e, **PROX1**, RGS4,**RRM2**, **STMN1,TCF4, TRO**,tubulin (complex),USP12,**ZEB2**
Cellular Assembly and Organization, Cellular Function and Maintenance, Cell Morphology	29	19	ABL1, ACKR2, CEBPA, DIDO1, **DNER, DPYSL5, EEF1A2, EFNA5**, ERK1/2, EXTL2 ,FOXO1, GRB2, **GRIP1**, **HIST1H2AH** ,HNRNPA2B1, **KIF18A, LMNB1,LRRN1,LY6H, MAP2**,NUPR1,PFKFB4, **PFN2**,PLOD2, RHOA,**RIMKLA ,RNF144A, SLC32A1,SPAG5, SPC25,SYT1**, UBE2I,UHRF2, **WASF1**,WASF3
Nervous System Development and Function, Organ Morphology, Organismal Development	20	11	**AHI1, BEX1, CACNG5, CHGB, CPLX1, CRMP1, DCX, GAP43, GJD2**, HAP1, IGF1, KIF1A, **PAK3,** PDX1, PPP1R1A,REST,SBDS,**SYN1**

Supplementary Table S3: Genes up-regulated in Early NE samples: top canonical pathways

Ingenuity Canonical Pathways	-log(*p*-value)	Ratio	Molecules
Semaphorin Signaling in Neurons	3.92	0.0962	DPYSL2,CRMP1,PAK3,DPYSL4,DPYSL5
Axonal Guidance Signaling	2.66	0.0259	DPYSL2,KLC1,TUBA1A,PAK3,EPHB2,BDNF,EFNA5,PFN2,DPYSL5,ROBO1,TUBB2B
Notch Signaling	2.31	0.0811	CNTN1,MFNG,HEY1
Cyclins and Cell Cycle Regulation	2.24	0.0519	MYT1,CCNE2,E2F2,CCNB1
GADD45 Signaling	1.88	0.105	CCNE2,CCNB1

Supplementary Table S4: Genes overexpressed in human NE tumors enriched in OPT-tm vs T30 comparison: functional network analysis

Top Network	Score	Focus Protein	Molecules
Cell Cycle, DNA Replication, Recombination, and Repair, Cell Death and Survival	54	31	**CCNA2,CDC6,CDC7,CDT1**,Cyclin A,Cyclin E,**DTX3, DUSP14,E2f,E2F1,E2F2,FEN1,FOXM1,IL33, MALT1, MCM2,MCM5,MCM7, MCM10, MYBL2,NAMPT**,NFkB (complex)**, NUSAP1, PKMYT1,PLK1,PLK4,PRR11,RRM2 ,SENP1**,SKP2,**TPX2,TSLP,WDHD1,WDR76,ZC3H12A**
Cell Cycle, DNA Replication, Recombination, and Repair, Cell Death and Survival	35	23	**ASAH1,ASF1B**,AURKA,**BMP4,BUB1B,C1QTNF9,CDC20,CDC42EP5,CDCA5,DUSP4,ENO2**,ERK,ERK1/2,Histone h3,Hsp90,IFN Beta,IgG,Interferon alpha,Jnk,**LMNB1,MAPK8IP1,NCAPD2,NTRK3,OAS2**,P38 MAPK,PI3K (complex),**PRKCH,STK17B,SUV39H1,TCR,TNFRSF13C,TOP2A,UBE2S**,Vegf,**WT1**
Cancer, Skeletal and Muscular Disorders, Cell Cycle	23	19	**ASPM**,AURKB,BLM,CCND1,CD24,**CDCA8**,CDK4,**CENPI, CKAP2L,DPYSL5,DUSP8**,E2F8, FOXO1, GLI1,**INCENP,KIF23,MAN2B2**,NUPR1,**PLK1,PSTPIP2,SHCBP1**,SPC24,**SPC25,SUOX**, TGFB1,**XRCC2,ZMYM3**
Cell Cycle, DNA Replication, Recombination, and Repair, Cancer	19	21	AKAP12,**ANLN,ANPEP**,BRCA1,**CCNE2**,CD24,CDKN1A,CEBPA,**CENPA,CHAF1A,CHAF1B, CLSPN**, CNOT6,CNOT6L,CSF2,**DEPDC1B**,E2F4,**EEF1A2,FEN1**, FOXM1,HCAR2,HERC2,**IQGAP3, KIAA0101,KIF14,KNTC1**,NPAT,ORC1,**PLK1,PRC1**, PSMA3,RBBP8,SMC4,SRSF2,**TOP2A**

Supplementary Table S5: Genes overexpressed in human NE tumors enriched in *Early NE vs Early ADC* comparison: functional network analysis

Top Network	Score	Focus Protein	Molecules
Cancer, Organismal Injury and Abnormalities, Reproductive System Disease	59	31	**AURKA,BMP4,BUB1B,CCNA2,CDC6,CDC7,CDC20,CDCA4,CDT1,CENPA,CEP55**,Cyclin A, Cyclin E,**DUSP14**,E2f,**E2F1,E2F2,FOXM1,GTSE1,KIF11,KIF23,LACTB,MCM5,MCM10 ,MYBL2**,NFkB (complex)**,NUSAP1,PLK1,PLK4,PRR11,RRM2,SKP2,TK1,TPX2,WDR76**
DNA Replication, Recombination, and Repair, Cell Cycle, Cellular Assembly and Organization	29	19	AKAP12,**ASF1B,CDC37L1,CDC42EP5,CDT1** ,Cg**,DAZAP1,DUSP3,DUSP14,ENO2**,ERK1/2**, ESM1**,FKBP4,FSH,Histone h3,Hsp90,IGFBP5,Interferon alpha,Lh ,MAP3K5**,MAPK8IP1,NCAPD2**,P38 MAPK**,PRKCH,RAB27A**,SMC2, SMC4**,SRD5A2**,STIP1**,SUV39H1,TK1,TLK1,TOP2A,WDHD1**,YBX3
Hematological System Development and Function, Hematopoiesis, Tissue Development	25	17	AGTR1,**ANPEP,AP3B2,BMP4**,CEBPA,CENPF,**CHAF1B**,CNOT6,CNOT6L,CSF2,**EEF1A2,ESPL1**, FLT1,G3BP1,GAB1,**GIPC2,GNG4**,GRB2,HAMP,HNRNPD,ILF2,**ILF3,L3MBTL3,LHX2,MAP1B**,mir-181,NANOG,**NKX2-2,RAD51AP1,RRAS**,SEMA3A,SP1,**SYP**,TGFB1,**TOP2A**
DNA Replication, Recombination, and Repair, Cell Cycle, Cell Morphology	21	15	ASPM,ATR,BRCA1,BRCA2,CCNK,CDCA8,CENPI,CHEK1,CKAP2L,CLSPN,DPYSL5,EIF4G1,FANCD2,HERC2,LMNB1,MAN2B2,MBD2,MCM2,miR-483-3p (miRNAs w/seed CACUCCU),MSH2,NBN,NDC80,NUF2,NUPR1,PLK1,RAD51,RFC4,RFC5,RNF168,RPA1,RPA2,SHCBP1,SPC24,SPC25,SUOX
DNA Replication, Recombination, and Repair, Cell Cycle, Cancer	21	15	ANLN,C1QTNF9,CALCR,CCNK,CDCA5,CHRNA3,CHRNB2,DSCC1,DUT,E2F4,ERK,FOXO3,HMMR,IGF1,KIAA0101,Mek,MYBL2,POLA2,POLD1,POLD2,PPP1R13B,PRKAA2,PRKAB1,PRKAG1,RAMP1,RFC3,RPS27L,RRM2,SMC4,SYN1,TIMELESS,TOP2A,TP53,UBE2T,UNG
Cell Cycle, Cell Death and Survival, Cancer	17	13	ALDH1A2,ATF3,BCL2L14,BICD2,BRD4,CD24,CDC25A,CDKN1A,CFTR,CUL1,DEPDC1B,FADS1,H2AFX,IL13,KIF2A,KNTC1,LRPPRC,MANEAL,NAV1,NME1,NR3C1,NRIP1,PCM1,PDLIM2,POLR2A,SALL2,STX1A,STXBP1,THBS1,TOP2A,TP53BP2,YWHAG,YWHAQ,YWHAZ,YY1
